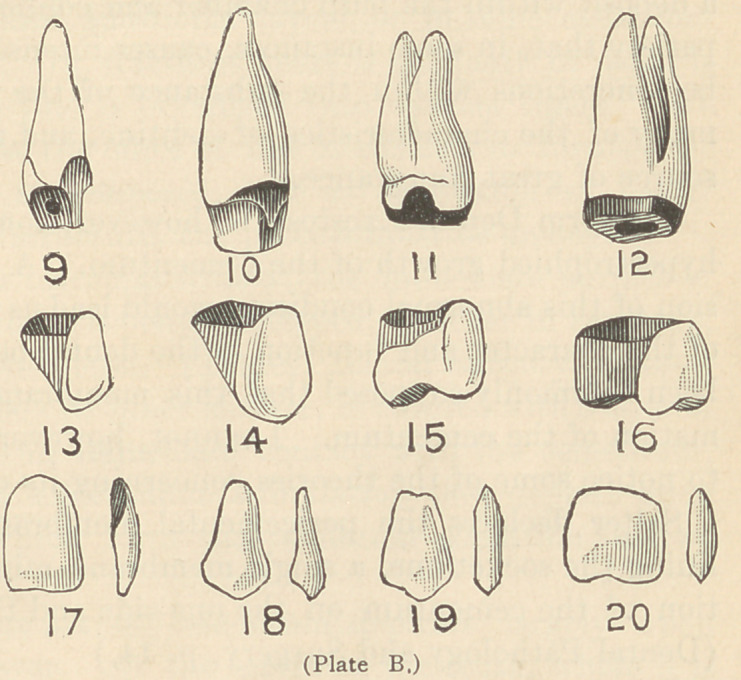# A New System of Restoring Badly Decayed Teeth by Means of an Enameled Metallic Coating

**Published:** 1886-08

**Authors:** C. H. Land

**Affiliations:** Detroit, Mich.


### A NEW SYSTEM OF RESTORING BADLY DECAYED TEETH BY MEANS OF AN ENAMELED METALLIC COATING.

BY DR. C. H. LAND, DETROIT, MICH.

This invention consists of an artificial coating of platinum made to fit the outside of the teeth, after which the anterior surface is coated with a porcelain enamel front, made to imitate the natural organs so perfectly that the art is concealed. Many of the long and tedious operations, where it has been deemed necessary to insert large and conspicuous gold fillings, may, by this process, be avoided, while better results are attained.

(Fig. 21.)

Fig. 21 is a typical case, where, in place of inserting the usual gold fillings, the anterior surface may be reduced by means of small corundum wheels used in the dental engine, as indicated in Figures 1 and 2, Plate A. Fig. 13, Plate B, is the prepared crown ready for adjustment to the same by the use of oxy-phosphate cement. Fig. 22 represents a typical case of undeveloped lateral incisors, which can be enlarged to their proper size by the same means.

Fig. 4, Plate A, represents a decayed molar. Fig. 8 is the same prepared to receive the amalgam filling which, when sufficiently hard, is prepared as shown in Fig. 12, ready to have the crown, Fig. 16, cemented to it with oxy-phosphate cement. Fig. 9 is a central incisor, Fig. 10 a cuspid, and Fig. 11 a bicuspid. Figures 13, 14 and 15 the crowns ready for adjustment. Those who object to the use of amalgam may use white cement or gutta-percha for fastening.

The manner of procedure in the case of devitalized and discolored incisors is first to prepare the teeth as shown in Plate A, Figs. 1 and 2. Then a thin piece of platinum plate, No. 30 standard gauge, should be fitted accurately to the tooth, forming a hollow shell. Enamel fronts are now ground to fit, as shown in Figs. 17, 18, 19, and 20, after which they are fused to the platinum in the same manner as continuous gum work, by using a porcelain body prepared expressly for the purpose. By the use of

(Fig. 22.)

Land's Gas Furnace this can be done in ten minutes. The enamel fronts and body are also manufactured and for sale by the Wilmington Dental Manufacturing Co.

Fig. 9 represents a central incisor built up with amalgam or cement, to which the platinum is closely fitted, after which the enamel front, Fig. 17, is ground to fit and fused to the shell, as shown in Fig. 13, ready for adjustment to Fig. 9. Figs. 10, 11, and 12 are modifications for canine, bicuspid, and molars, ready to receive the prepared coatings, Nos. 14, 15, and 16.

(Plate A.)

In introducing this class of work to the dental profession a means is offered through which a much better artistic effect can be attained and the preservation of a larger amount of tooth structure be secured. Add to this the fact that there is but very little pain or fatigue, either for the patient or operator, and it will be doubly appreciated.

During the past year this class of work has been thoroughly tested as to durability, and found to be much more reliable than gold fillings. In large contour work the frail walls of the tooth must be the main dependence of support, while with the hollow shell the weak tooth is held together. Thus it will be seen how much more complete is the preservation of tooth substance, it not being necessary to make undercuts or retaining pits.

A young lady recently presented herself with both central incisors broken off by an accident, the left one having lost about half its crown, with complete exposure of the pulp; the right one having only about one-sixth of its substance gone. Her teeth were unusually well preserved, while they were large and quite conspicuous. The right central was easily restored to a good contour by a little grinding. The left, after necessary treatment, was simply ground down upon the anterior surface, an operation demanding less than ten minutes of time. In twenty minutes more a platinum overcoat was fitted to it and the enamel front ground to fit. This completed the first sitting. In the afternoon of the same day it required but a sitting of fifteen minutes to adjust the prepared coating. The result was a complete restoration with the least possible amount of inconvenience to the patient, and the greatest amount of tooth substance preserved. Contrast this operation with what it would have been necessary to do had I attempted to restore the tooth by means of a gold filling, or to place upon the root a properly prepared gold crown. Think of the long and tedious operation, and when completed what a conspicuous piece of mouth jewelry it would have presented, and you may, perhaps, realize a part of the degree of satisfaction which I felt when I had finished my operation. This, together with a series of many similar cases, may form part of a future illustrated article.

## Figures and Tables

**Fig. 21. f1:**
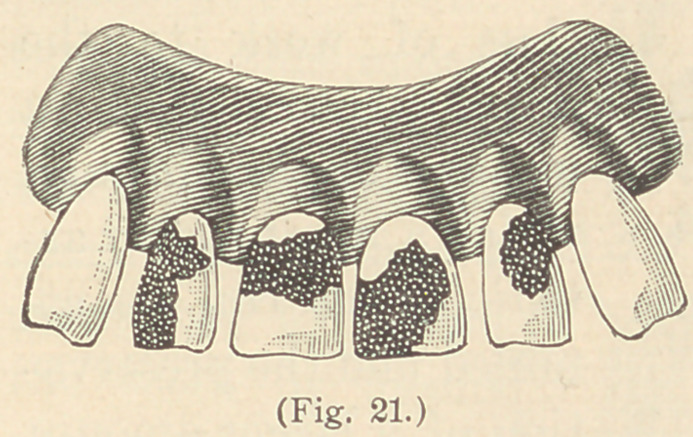


**Fig. 22. f2:**
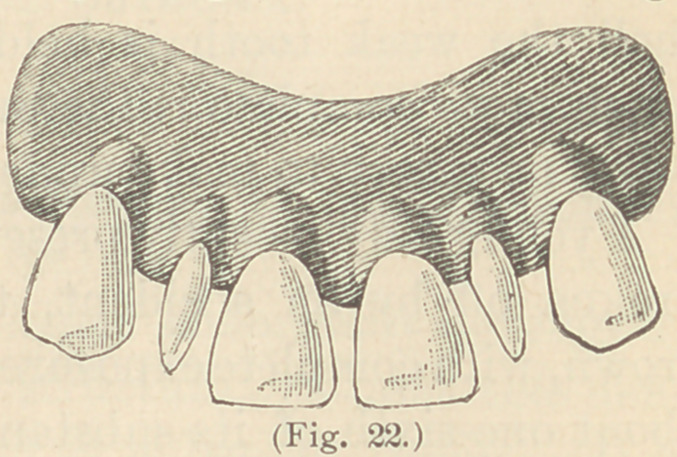


**Plate A. f3:**
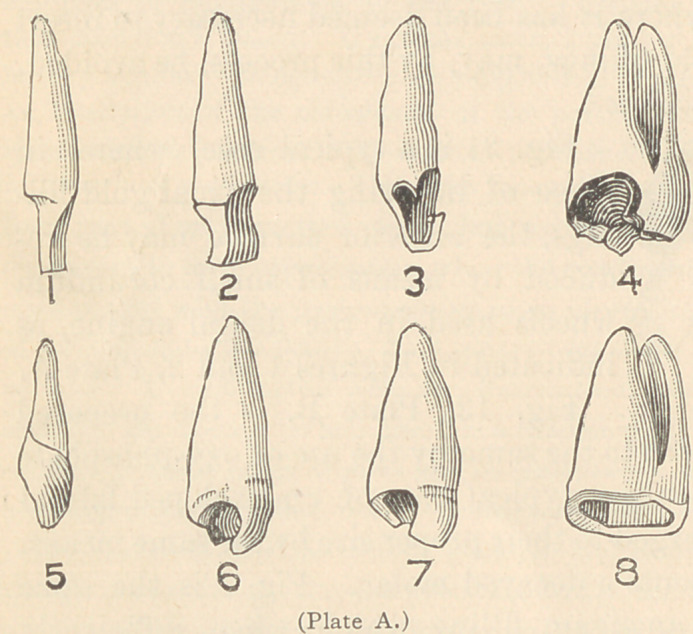


**Plate B. f4:**